# Supplementary material for: Synapse type-specific proteomic dissection identifies IgSF8 as a hippocampal CA3 microcircuit organizer
Source: Nat Commun. 2020 Oct 14;11:5171. doi: 10.1038/s41467-020-18956-x (PMC7560607; doi:10.1038/s41467-020-18956-x)
Supplement: Supplementary file 1 — Supplementary Info [file 41467_2020_18956_MOESM1_ESM.pdf]

Supplementary Figures and Supplementary Figure Legends

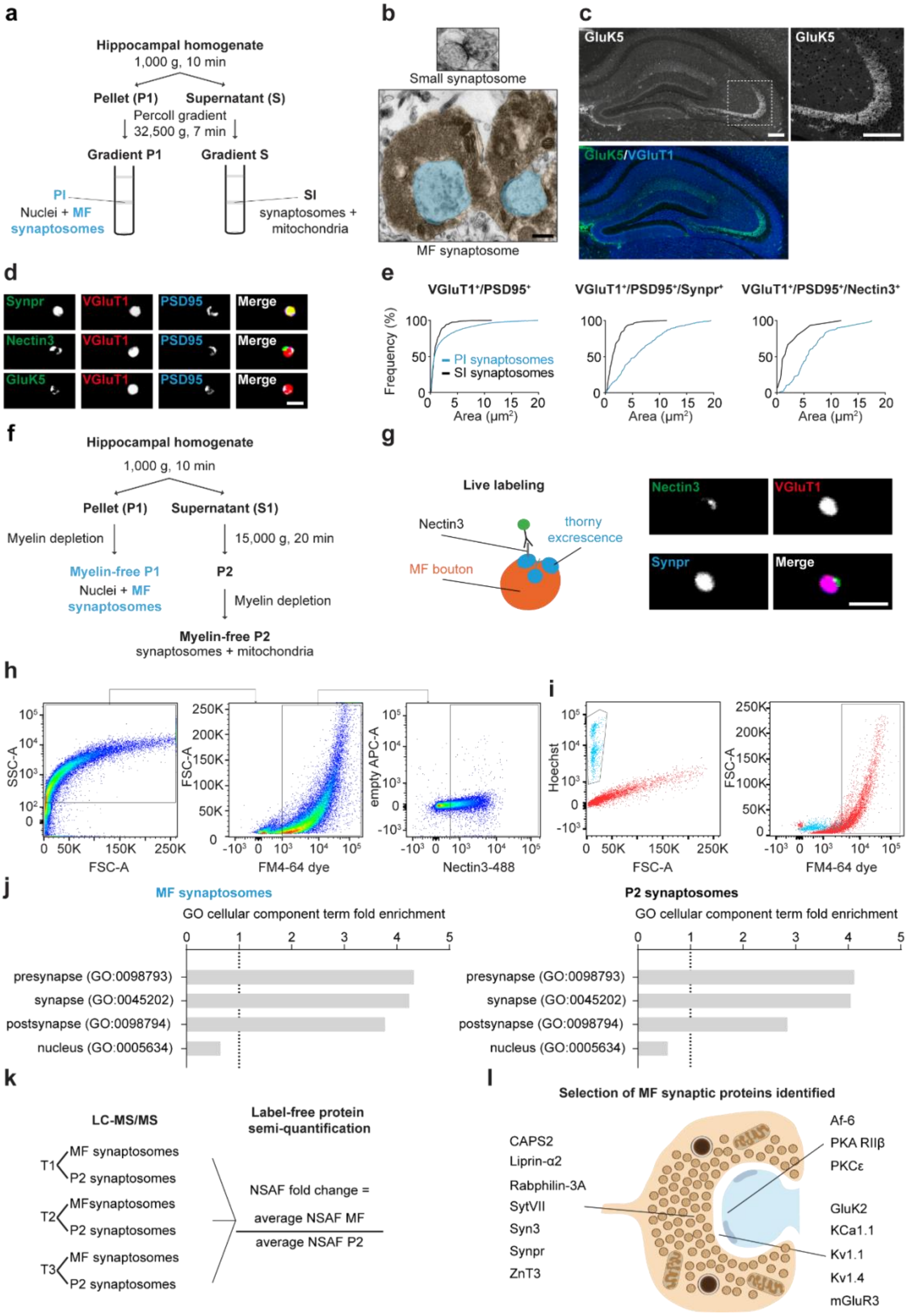

Supplementary Figure 1. Isolation and proteomic profiling of MF synaptosomes. (**a**) Original method to isolate MF synaptosomes from mouse hippocampal homogenates using Percoll gradients[1]. (**b**) Electron microscopy image of a small synaptosome (top) and a large MF synaptosome (bottom) detected in PI, displayed at same scale for comparison. Large MF bouton highlighted in orange filled with synaptic vesicles and containing mitochondria. Postsynaptic spines highlighted in blue. (**c**) Confocal images of P28 mouse hippocampal sections immunostained for GluK5 and VGluT1. Magnified inset of SL in CA3 is shown on the right. (**d**) Confocal images of MF synaptosomes captured in PI immunostained for Synpr, Nectin-3, GluK5, VGluT1 and PSD95. Nectin 3 labeling was confined to small puncta as expected for a PA-localized postsynaptic protein. (**e**) Area distribution of VGluT1^{+}/PSD95^{+}, VGluT1^{+}/PSD95^{+}/Synpr^{+} and VGluT1^{+}/PSD95^{+}/Nectin3^{+} synaptosomes in PI and SI. (**f**) Optimized method for biochemical enrichment of MF synaptosomes starting from P28 mouse brains. (**g**) Live labelling strategy (left) and confocal images (right) of MF synaptosomes live-labeled with an Alexa 488-conjugated anti-Nectin-3 monoclonal antibody. (**h**) Gating strategy to sort FM4-64/Nectin3-488 double-labeled MF synaptosomes. (**i**) Nuclei labeled with Hoechst are not labeled by FM4-64 dye. (**j**) GO analysis on all proteins detected in sorted MF synaptosomes or P2 synaptosomes. Fold enrichment of a selection of significant cellular component terms. (**k**) Outline of the experimental set-up for the LC-MS/MS analysis of sorted MF synaptosomes and P2 synaptosomes. NSAF FC was calculated using the average NSAF of 3 independent experiments. (**l**) Cartoon illustrating a selection of known MF synaptic proteins detected in isolated MF synaptosomes. Source data are provided as a Source Data file. Scale bars in (b) 0.5 μm, in (c) 200 μm, and in (d) and (g) 5 μm.

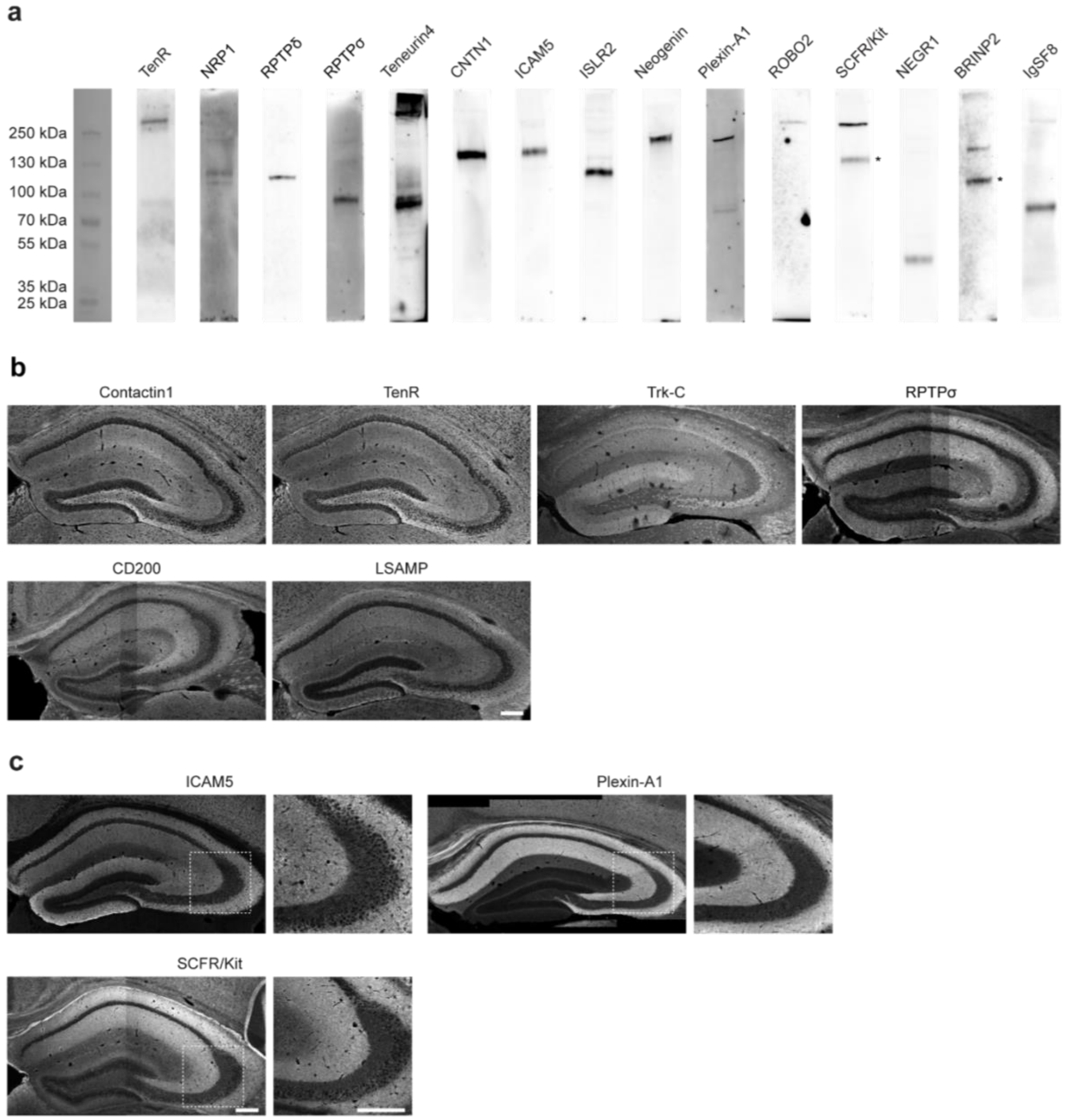
a
TenR
NRP1
RPTPδ
RPTPσ
Teneurin4
CNTN1
ICAM5
ISLR2
Neogenin
Plexin-A1
ROBO2
SCFR/Kit
NEGR1
BRINP2
IgSF8
250 kDa
130 kDa
100 kDa
70 kDa
55 kDa
35 kDa
25 kDa
b
Contactin1
TenR
Trk-C
RPTPσ
CD200
LSAMP
c
ICAM5
Plexin-A1
SCFR/Kit

Supplementary Figure 2. Dissection of MF synapse CSP composition. (**a**) Selection of CSP antibodies working in WB using mouse hippocampal lysates. Asterisks indicate bands of expected size for respective CSPs. (**b**) Confocal images of P28 mouse hippocampal sections immunostained for 6 CSPs detected in sorted MF synaptosomes showing broad expression in the hippocampus, including SL. (**c**) CSPs validated to be present in MF synaptosomes by WB (Fig. 2d) but with little immunoreactivity in SL. Insets show high-magnification images of the SL. Images in (b) and (c) represent the best labelling obtained in multiple conditions tested (Supplementary Data 2). Scale bars in (b) and (c) 200 μm.

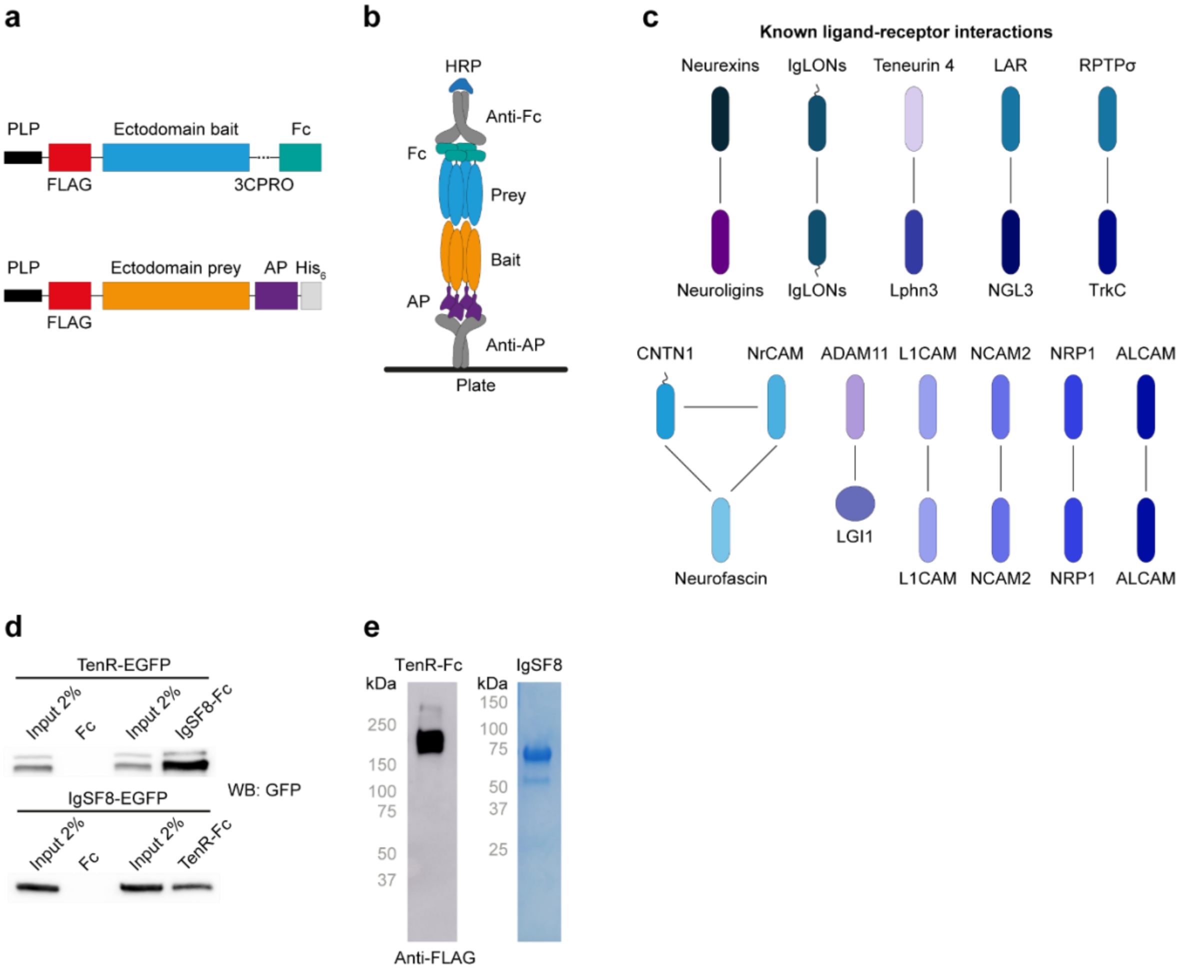
a
PLP
Ectodomain bait
Fc
FLAG
3CPRO
PLP
Ectodomain prey
AP His6
FLAG
b
HRP
Anti-Fc
Fc
Prey
Bait
AP
Anti-AP
Plate
c
Known ligand-receptor interactions
Neurexins
IgLONs
Teneurin 4
LAR
RPTPσ
Neuroligins
IgLONs
Lphn3
NGL3
TrkC
CNTN1
NrCAM
ADAM11
L1CAM
NCAM2
NRP1
ALCAM
LGI1
Neurofascin
L1CAM
NCAM2
NRP1
ALCAM
d
TenR-EGFP
Input 2%
Fc
Input 2%
IgSF8-Fc
WB: GFP
IgSF8-EGFP
Input 2%
Fc
Input 2%
TenR-Fc
e
TenR-Fc
IgSF8
kDa
250
150
100
75
50
37
kDa
150
100
75
50
37
25
Anti-FLAG

Supplementary Figure 3. MF synapse CSP interactome screening. (**a**) Schematic diagram of the constructs used to perform the interactome analysis. Ectodomains of transmembrane and GPI-anchored proteins, or full-length protein in case of secreted proteins, were C-terminally tagged with human Fc fragment or alkaline phosphatase (AP). Both constructs have a leader peptide (PLP) to ensure that recombinant proteins are secreted. The Fc-fusion has a 3CPro cleavage site for purification purposes, whereas the AP-fusion construct has an additional His tag. (**b**) Schematic diagram of the ELISA-based assay showing the orientation of bait and prey recombinant proteins. (**c**) Interaction networks of known ligand-receptor pairs identified including NRXNs-NLGNs and the IgLONs. (**d**) Pull-down assays in transfected HEK293T cells show IgSF8-TenR interaction. Representative WBs from two independent experiments. (**e**) WB of conditioned medium of HEK293 cells transfected with TenR-Fc and Coomassie blue staining of purified IgSF8 used in Biolayer interferometry experiment. Source data are provided as a Source Data file. Scale bar in (d) 5 μm.

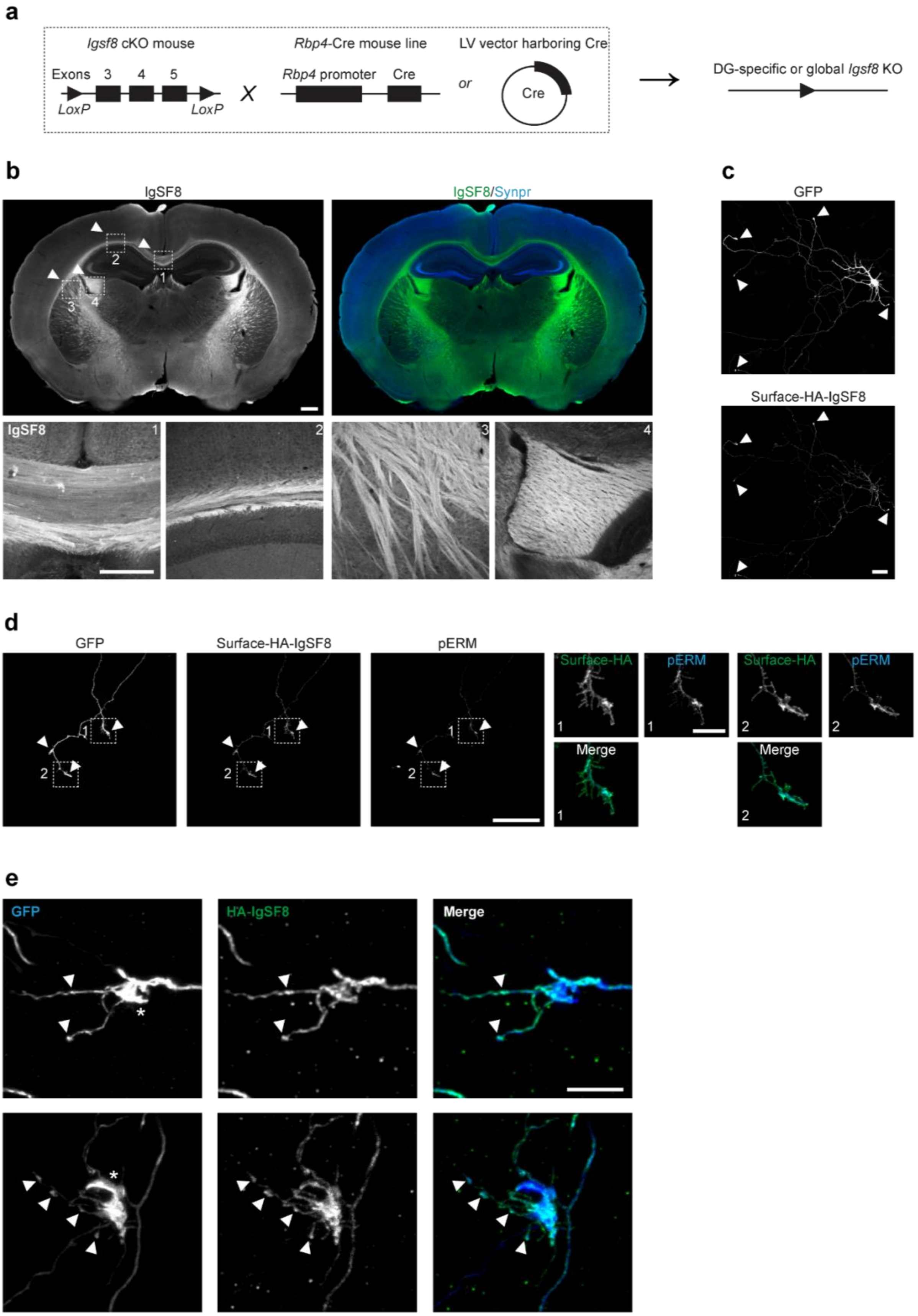

a
Igsf8 cKO mouse
Exons 3 4 5
LoxP
LoxP
X
Rbp4-Cre mouse line
Rbp4 promoter Cre
or
LV vector harboring Cre
Cre
DG-specific or global Igsf8 KO
b
IgSF8
IgSF8/Synpr
1
2
3
4
c
GFP
Surface-HA-IgSF8
d
GFP
Surface-HA-IgSF8
pERM
Surface-HA
pERM
Merge
e
GFP
HA-IgSF8
Merge

Supplementary Figure 4. IgSF8 localizes to MF boutons and filopodia. (**a**) Experimental setup to delete *Igsf8 in vivo* and *in vitro* using the *Igsf8* cKO mouse and the *Rbp4*-Cre line or an LV vector harboring Cre recombinase, respectively. (**b**) Wide field microscopy imaging of a P28 mouse brain section highlights prominent IgSF8 labeling in fiber tracts in addition to the hippocampal MFs. Magnified insets show IgSF8 labeling of fiber tracts in the corpus callosum (insets 1 and 2), the internal capsule (inset 3) and fimbria (inset 4). (**c**) Confocal low-magnification images of cultured primary mouse cortical neurons expressing the recombinant construct GFP-T2A-HA-IgSF8 in the FUGW backbone plasmid. In this construct IgSF8 is N-terminally tagged with the HA epitope. Neurons were fixed between DIV 7-8 and immunostained for HA before permeabilization to analyse IgSF8 surface localization, and for GFP after permeabilization to help visualizing neuronal layout. Surface HA-tagged IgSF8 localizes preferentially to extending terminals of neuronal processes including axonal growth cones (indicated with arrowheads). (**d**) Primary mouse cortical neurons cultured in similar conditions as in (c). Higher-magnification images show surface HA-tagged IgSF8 in extending growth cones (arrowheads), where it co-localizes with pERM. Magnified insets on the right show growth cones marked with arrowheads. Surface HA-tagged IgSF8 is shown in green while pERM is shown in blue in merged images, highlighting surface HA-IgSF8 co-localization with pERM. (**e**) Additional examples of stacks of confocal images showing mGFP-labelled MF boutons and respective filopodia (in blue in merged images). HA-IgSF8 is concentrated in the MF bouton but also observed in defined regions along the filopodia including their terminals (in green in merged images). Representative images from at least two independent experiments from (b) to (e). Scale bars in (b) 400 µm, in insets of (b) 200 µm, in (c) and (d) 50 µm, in insets of (d) and (e) 10 µm.

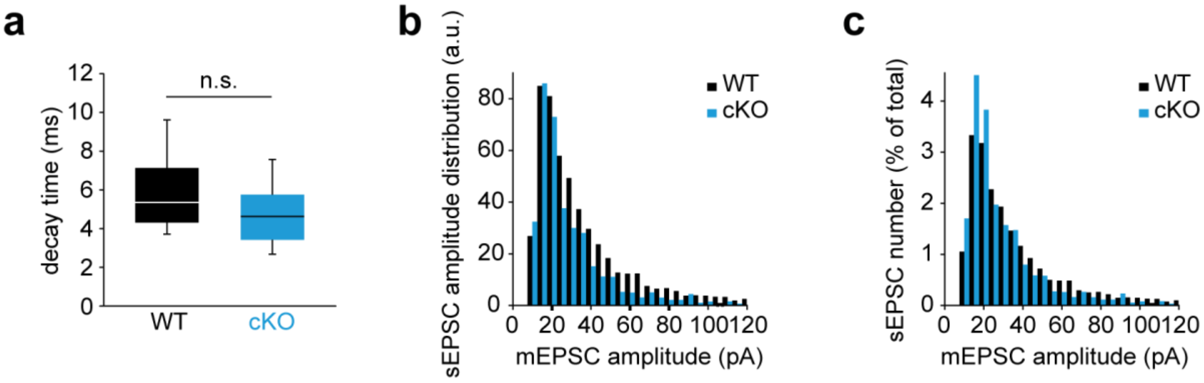

a
n.s.
decay time (ms)
12
10
8
6
4
2
0
WT
cKO
b
sEPSC amplitude distribution (a.u.)
WT
cKO
80
60
40
20
0
0 20 40 60 80 100120
mEPSC amplitude (pA)
c
sEPSC number (% of total)
WT
cKO
4
3
2
1
0
0 20 40 60 80 100120
mEPSC amplitude (pA)

Supplementary Figure 5. Loss of IgSF8 impairs spontaneous synaptic transmission in CA3 neurons. (**a**) Quantification of sEPSC decay time. (**b**) Histogram analysis of sEPSC amplitude distributions in *Rbp4-Cre:Igsf8* cKO and WT littermates. (**c**) Histogram analysis of sEPSC amplitude distributions (% of total) in *Rbp4-Cre*:*Igsf8* cKO and WT littermates. WT and cKO littermate mice were examined over three independent experiments (WT, n = 31 neurons and cKO, n = 38). Box-and-whisker plot in (a) shows median, interquartile range, minimum and maximum. Mann-Whitney test was used in (a). n.s., not significant. Source data are provided as a Source Data file.

**pEGFP-N1-Igsf8**
Igsf8 Forward - ATTATTAAGCTTATGGGCGTCCCTAGCCCCACG
IgSF8 Reverse - ACTACTGTCGACTTCCGCTTCCGCATCCTCTTC
**pEGFP-N1-Tenascin-R**
Tenascin-R Forward - CGTCGTAAGCTTATGGGGATCGATGGGGAAAC
Tenascin-R Reverse - ACTACTGTCGACTTGAATTTCAAGGCTCGCCG
**Brevican-Fc**
FLAG-Brevican Forward - CGATGACGACAAGGCGGCCGCGGATGACCTGAAAGAAGACAGC
Ig-Brevican Reverse - GCACCTCTAGACCAGTCGACAAGAGACTGGAGGGCGGT
**FUGW-mGFP-T2A-HA-IgSF8**
mGFP Forward - GGCTGCAGGTCGACTCTAGAGGATCCATGGGTTGCTGTTTCTCC
mGFP-T2A-SP-HA-Igsf8 Reverse - AGCGTAATCTGGAACGTCATATGGATAGGCGTAGCACCTGGTTCCAAGTATTAGCAGCAGCAGCAGGAGCGAACTCAGCGGCGTGGGGCTAGGGACGCCCATGGGCCCAGGATTCTCCTCGACGTCACCGCATGTTAGCAGACTTCCTCTGCCCTCTCCACTGCCCTTGTACAGCTCGTCCATGCC
HA-Igsf8 Forward - TATGACGTTCCAGATTACGCTCGGCAGGTGCATGTCCCCAGG
Igsf8 Reverse - CGATAAGCTTGATATCGAATTCTTACCGCTTCCGCATCCTCTTCAT
**FUGW-GFP-T2A-HA-IgSF8**
GFP Forward - TTAGACGAAGCTTGGGCTGCAGGTCGACTCTAGAGGATCCGCCACCATGGTGAGCAAG
GFP-T2A Reverse - TAGCAGACTTCCTCTGCCCTCTCCACTGCCGAATTCCTTGTACAGCTCGTCCATG
GFP-T2A Forward - GGGATCACTCTCGGCATGGACGAGCTGTACAAGGAATTCGGCAGTGGAGAGGGCAGA
Igsf8 Reverse - TCCAGAGGTTGATTATCGATAAGCTTGATATCGAATTCTTACCGCTTCCGCATCCTC
**pAAV-hSyn1-mGFP**
hSyn1-mGFP Forward - CCTGAGAGCGCAGTCGAGAGGATCCGCCACCATGGGTTGCTGTTTC
WPRE-mGFP Reverse - CCAGAGGTTGATTATCGATAAGCTTTTACTTGTACAGCTCGTCCATGC
**pAAV-hSyn1-EGFP-T2A-CRE**
hSyn1-EGFP Forward - CCTGAGAGCGCAGTCGAGAGATGGTGAGCAAGGGCGAG
WPRE-CRE-Reverse - CCAGAGGTTGATTATCGATACTAAGCCATCTGCAGCTG
**pAAV-hSyn1-mGFP-T2A-CRE**
T2A Forward - AAGCTTGAGGGCAGAGGCTCCCTG
hSyn1 Reverse - GAATTCCTCTCGACTGCGCTCTCAG
hSyn1-mGFP Forward - CCTGAGAGCGCAGTCGAGAGGAATTCGCCACCATGGGTTGCTGTTTC
T2A-mGFP Reverse - AGCAGGGAGCCTCTGCCCTCAAGCTTCTTGTACAGCTCGTCCATGC

Supplementary Table 1. List of primers. List of oligonucleotides used to clone the DNA constructs relevant for the study.
