# Supplementary material for: Synapse type-specific proteomic dissection identifies IgSF8 as a hippocampal CA3 microcircuit organizer
Source: Nat Commun. 2020 Oct 14;11:5171. doi: 10.1038/s41467-020-18956-x (PMC7560607; doi:10.1038/s41467-020-18956-x)
Supplement: Supplementary file 3 — Description of Additional Supplementary Files [file 41467_2020_18956_MOESM3_ESM.pdf]

Description of Additional Supplementary Files

Title: Supplementary Data 1

Description: Proteomic profiling of MF synaptosomes. (Tab1) Raw data file containing all proteins detected in 3 independent experiments in MF synaptosomes and P2 synaptosomes. (Tab2) All proteins detected with at least 3 peptide identifications among 3 independent experiments in MF synaptosomes and P2 synaptosomes. (Tab3) Significant proteins with positive sorted MF synaptosomes/P2 synaptosomes FC or exclusively detected in sorted MF synaptosomes. (Tab4) All significant CSPs with positive sorted MF synaptosomes/P2 synaptosomes FC or exclusively detected in sorted MF synaptosomes. (Tab5) Significant CSPs with positive sorted MF synaptosomes/P2 synaptosomes FC. (Tab6) Significant CSPs exclusively detected in sorted MF synaptosomes.

Title: Supplementary Data 2

Description: Dissection of MF synapse CSP composition. (Tab1) Overview of CSPs detected in isolated MF synaptosomes and information on functional group; protein family/domain; previously reported association with MF synapses or synapses in general; and annotation in SynGO. (Tab2) Validation of a selection of CSPs by WB and IHC.

Title: Supplementary Data 3

Description: MF synapse CSP interactome screening. (Tab1) Overview of AP- and Fc-tagged recombinant proteins used in each independent experiment. (Tab2) Total ligand-receptor interaction pairs identified per experiment and globally in all three independent experiments. In total, 38 interaction pairs were confidently identified (detected at least twice, independently of the orientation). (Tab3) Global matrix displaying confidently detected pairwise interactions.

Title: Supplementary Data 4

Description: Ecto-Fc pull-downs. (Tab1) Spectral and peptide counts of proteins captured in 1 independent pull-down experiment using whole rat brain synaptosome lysates. (Tab2) Spectral counts of proteins captured in 2 independent pull-down experiments using mouse P1 MF synaptosome lysates.
