# Supplementary material for: Synapse type-specific proteomic dissection identifies IgSF8 as a hippocampal CA3 microcircuit organizer
Source: Nat Commun. 2020 Oct 14;11:5171. doi: 10.1038/s41467-020-18956-x (PMC7560607; doi:10.1038/s41467-020-18956-x)
Supplement: Supplementary file 8 — Reporting Summary [file 41467_2020_18956_MOESM8_ESM.pdf]

nature research

Corresponding author(s): De Wit, Joris

Last updated by author(s): Sep 10, 2020

Reporting Summary

Nature Research wishes to improve the reproducibility of the work that we publish. This form provides structure for consistency and transparency in reporting. For further information on Nature Research policies, see Authors & Referees and the Editorial Policy Checklist .

Statistics

For all statistical analyses, confirm that the following items are present in the figure legend, table legend, main text, or Methods section.

n/a	Confirmed	
☐	☒	The exact sample size (*n*) for each experimental group/condition, given as a discrete number and unit of measurement
☐	☒	A statement on whether measurements were taken from distinct samples or whether the same sample was measured repeatedly
☐	☒	The statistical test(s) used AND whether they are one- or two-sided<br>*Only common tests should be described solely by name; describe more complex techniques in the Methods section.*
☒	☐	A description of all covariates tested
☐	☒	A description of any assumptions or corrections, such as tests of normality and adjustment for multiple comparisons
☐	☒	A full description of the statistical parameters including central tendency (e.g. means) or other basic estimates (e.g. regression coefficient) AND variation (e.g. standard deviation) or associated estimates of uncertainty (e.g. confidence intervals)
☐	☒	For null hypothesis testing, the test statistic (e.g. *F*, *t*, *r*) with confidence intervals, effect sizes, degrees of freedom and *P* value noted<br>*Give P values as exact values whenever suitable.*
☒	☐	For Bayesian analysis, information on the choice of priors and Markov chain Monte Carlo settings
☒	☐	For hierarchical and complex designs, identification of the appropriate level for tests and full reporting of outcomes
☒	☐	Estimates of effect sizes (e.g. Cohen's *d*, Pearson's *r*), indicating how they were calculated

Our web collection on statistics for biologists contains articles on many of the points above.

Software and code

Policy information about availability of computer code

Data collection	RawConverter (http://fields.scripps.edu/downloads.php); ProLuCID/SEQUEST algorithm (ProLuCID ver. 3.1); DTASelect2 (ver. 2.1.3); through Integrated Proteomics Pipeline (IP2 v.3, Integrated Proteomics Applications, Inc., CA, USA http://www.integratedproteomics.com); Leica Application Suite X (LAS X, ver. 4.3 - Leica Microsystems)
Data analysis	FIJI (ver. 1.53c, NIH); Imaris (ver. 9, Bitplane); Microscope Imaging Browser (ver. 2.511, University of Helsinki); GraphPad Prism8 (ver. 8, GraphPad Software, Inc); MiniAnalysis program (ver. 6.0.7, Synaptosoft); Clampfit (ver. 10.7, Axon Instruments)

For manuscripts utilizing custom algorithms or software that are central to the research but not yet described in published literature, software must be made available to editors/reviewers. We strongly encourage code deposition in a community repository (e.g. GitHub). See the Nature Research guidelines for submitting code & software for further information.

Data

Policy information about availability of data

All manuscripts must include a data availability statement. This statement should provide the following information, where applicable:

- Accession codes, unique identifiers, or web links for publicly available datasets
- A list of figures that have associated raw data
- A description of any restrictions on data availability

UniProt mouse protein database (downloaded on 03-25-2014) (The UniProt Consortium 2015) (https://www.uniprot.org/); Panther Classification System using the mouse genome as reference (http://www.pantherdb.org/); PubMed (https://www.ncbi.nlm.nih.gov/pubmed/); Proteomic analysis of sorted MF synaptosomes and P2 synaptosomes is available in Proteome Exchange: PXD013492.

Field-specific reporting

Please select the one below that is the best fit for your research. If you are not sure, read the appropriate sections before making your selection.

- [x] Life sciences
- [ ] Behavioural & social sciences
- [ ] Ecological, evolutionary & environmental sciences

For a reference copy of the document with all sections, see nature.com/documents/nr-reporting-summary-flat.pdf

Life sciences study design

All studies must disclose on these points even when the disclosure is negative.

Sample size	For quantitative analysis, no sample size calculation was performed. Experiments were designed so that there were at least three independent biological replicates, as an accepted standard procedure in the field.
Data exclusions	No data was excluded from analysis.
Replication	Experiments were performed with at least three independent biological replicates, as an accepted standard procedure in the field.
Randomization	Samples were randomly allocated to experimental groups. Analysis of sorted MF synaptosomes and P2 synaptosomes was done in a balanced mix of male and female mice.
Blinding	Data collection and analysis for all experiments including quantitative measurements between different conditions (assessment of morphological and electrophysiological parameters at the mossy fiber synapse) were done blind to the conditions.

Behavioural & social sciences study design

All studies must disclose on these points even when the disclosure is negative.

Study description	*Briefly describe the study type including whether data are quantitative, qualitative, or mixed-methods (e.g. qualitative cross-sectional, quantitative experimental, mixed-methods case study).*
Research sample	*State the research sample (e.g. Harvard university undergraduates, villagers in rural India) and provide relevant demographic information (e.g. age, sex) and indicate whether the sample is representative. Provide a rationale for the study sample chosen. For studies involving existing datasets, please describe the dataset and source.*
Sampling strategy	*Describe the sampling procedure (e.g. random, snowball, stratified, convenience). Describe the statistical methods that were used to predetermine sample size OR if no sample-size calculation was performed, describe how sample sizes were chosen and provide a rationale for why these sample sizes are sufficient. For qualitative data, please indicate whether data saturation was considered, and what criteria were used to decide that no further sampling was needed.*
Data collection	*Provide details about the data collection procedure, including the instruments or devices used to record the data (e.g. pen and paper, computer, eye tracker, video or audio equipment) whether anyone was present besides the participant(s) and the researcher, and whether the researcher was blind to experimental condition and/or the study hypothesis during data collection.*
Timing	*Indicate the start and stop dates of data collection. If there is a gap between collection periods, state the dates for each sample cohort.*
Data exclusions	*If no data were excluded from the analyses, state so OR if data were excluded, provide the exact number of exclusions and the rationale behind them, indicating whether exclusion criteria were pre-established.*
Non-participation	*State how many participants dropped out/declined participation and the reason(s) given OR provide response rate OR state that no participants dropped out/declined participation.*
Randomization	*If participants were not allocated into experimental groups, state so OR describe how participants were allocated to groups, and if allocation was not random, describe how covariates were controlled.*

Ecological, evolutionary & environmental sciences study design

All studies must disclose on these points even when the disclosure is negative.

Study description	*Briefly describe the study. For quantitative data include treatment factors and interactions, design structure (e.g. factorial, nested, hierarchical), nature and number of experimental units and replicates.*
Research sample	*Describe the research sample (e.g. a group of tagged Passer domesticus, all Stenocereus thurberi within Organ Pipe Cactus National Monument), and provide a rationale for the sample choice. When relevant, describe the organism taxa, source, sex, age range and any manipulations. State what population the sample is meant to represent when applicable. For studies involving existing datasets,*

describe the data and its source.

Sampling strategy: Note the sampling procedure. Describe the statistical methods that were used to predetermine sample size OR if no sample-size calculation was performed, describe how sample sizes were chosen and provide a rationale for why these sample sizes are sufficient.

Data collection: Describe the data collection procedure, including who recorded the data and how.

Timing and spatial scale: Indicate the start and stop dates of data collection, noting the frequency and periodicity of sampling and providing a rationale for these choices. If there is a gap between collection periods, state the dates for each sample cohort. Specify the spatial scale from which the data are taken

Data exclusions: If no data were excluded from the analyses, state so OR if data were excluded, describe the exclusions and the rationale behind them, indicating whether exclusion criteria were pre-established.

Reproducibility: Describe the measures taken to verify the reproducibility of experimental findings. For each experiment, note whether any attempts to repeat the experiment failed OR state that all attempts to repeat the experiment were successful.

Randomization: Describe how samples/organisms/participants were allocated into groups. If allocation was not random, describe how covariates were controlled. If this is not relevant to your study, explain why.

Blinding: Describe the extent of blinding used during data acquisition and analysis. If blinding was not possible, describe why OR explain why blinding was not relevant to your study.

Did the study involve field work? ☐ Yes ☐ No

Field work, collection and transport

Field conditions: Describe the study conditions for field work, providing relevant parameters (e.g. temperature, rainfall).

Location: State the location of the sampling or experiment, providing relevant parameters (e.g. latitude and longitude, elevation, water depth).

Access and import/export: Describe the efforts you have made to access habitats and to collect and import/export your samples in a responsible manner and in compliance with local, national and international laws, noting any permits that were obtained (give the name of the issuing authority, the date of issue, and any identifying information).

Disturbance: Describe any disturbance caused by the study and how it was minimized.

Reporting for specific materials, systems and methods

We require information from authors about some types of materials, experimental systems and methods used in many studies. Here, indicate whether each material, system or method listed is relevant to your study. If you are not sure if a list item applies to your research, read the appropriate section before selecting a response.

Materials & experimental systems

n/a	Involved in the study	
☐	☒	Antibodies
☐	☒	Eukaryotic cell lines
☒	☐	Palaeontology
☐	☒	Animals and other organisms
☒	☐	Human research participants
☒	☐	Clinical data

Methods

n/a	Involved in the study	
☒	☐	ChIP-seq
☐	☒	Flow cytometry
☒	☐	MRI-based neuroimaging

Antibodies

Antibodies used:
Chicken anti-GFP Aves Labs Cat#: GFP-1010; RID: AB_2307313
Mouse anti-GFP clone B-2 Santa Cruz Cat#: SC-9996; RRID: AB_627695
Mouse anti-c-myc clone 9E10 Santa Cruz Cat#: SC-40; RRID: AB_627268
Rabbit anti-6x-His Tag Thermo-Fisher Cat#: PA1-983B;
RRID: AB_1069891
Goat anti-MBP clone D-18 Santa Cruz Cat#: SC-13912;
RRID: AB_648794
Mouse anti-PSD95 clone 7E3-1B8 Thermo-Fisher Cat#: MA1-046;
RRID: AB_2092361
Rabbit anti-Histone H3 clone D1H2 Cell Signaling Technology Cat#: 4499 RRID: AB_10544537
Rabbit ant-Synaptophysin clone SVP-38 Sigma-Aldrich Cat#: S5768

RRID: AB_477523
Rat anti-Nectin3 clone 103-A1 Hycult Biotech Cat#: HM1053
RRID: AB_533278
Rabbit anti-Nectin3 Abcam Cat#: AB63931
RRID: AB_1142394
Mouse anti-GluK5/KA2 clone N279B/27 NeuroMab Cat#: 75-362
RRID: AB_2315855
Rabbit anti-Synaptoporin Synaptic Systems Cat#: 102 002
RRID: AB_887841
Guinea pig anti-VGluT1 Merck Millipore Cat#: AB5905
RRID: AB_2301751
Rabbit anti-Synapsin3 Synaptic Systems Cat#: 106 303
RRID: AB_2619775
Rabbit anti-Afadin clone A7L9H48 Thermo-Fisher Cat#: 700193
RRID: AB_2532299
Chicken anti-ZnT3 Synaptic Systems Cat#: 197 006
RRID: AB_2725754
Rabbit anti-mGluR2/3 Merck Millipore Cat#: AB1553
RRID: AB_11212089
Rabbit anti-CRTAC1 Merck Millipore Cat#: ABD80
Rabbit anti-BRINP2 Atlas Antibodies Cat#: HPA061920 RRID: AB_2684639
Goat anti-IgSF8 R&D Systems Cat#: AF3117
RRID: AB_2233385
Rabbit anti-FAM171A2 ThermoFisher Cat#: PA5-71105
RRID: AB_2689922
Rabbit anti-RPTPd Novus Cat#: NBP2-49153
Goat anti-RPTPs R&D Systems Cat#: AF3430
RRID: AB_2175157
Sheep anti-Teneurin-4 R&D Systems Cat#: AF6320
RRID: AB_10920937
Mouse anti-PSA-NCAM1 Merck Millipore Cat#: MAB5324
RRID: AB_95211
Rat anti-NCAM2 R&D Systems Cat#: MAB778
RRID: AB_2149709
Sheep anti-ISLR2 R&D Systems Cat#: AF4650
RRID: AB_2126610
Goat anti-NEGR1/Kilon R&D Systems Cat#: AF5394
RRID: AB_2150086
Mouse anti-Neuronal pentraxin 1 BD Biosciences Cat#: 610369
RRID: AB_397754
Goat anti-NRP1 R&D Systems Cat#: AF566
RRID: AB_355445
Goat anti-NRP2 R&D Systems Cat#: AF2215
RRID: AB_2155371
Sheep anti-Noelin R&D Systems Cat#: AF4636
RRID: AB_2157225
Goat anti-Plexin-A3 Thermo-Fisher Cat#: PA5-47571
RRID: AB_2608296
Rabbit anti-ROBO2 Aviva Systems Cat#: ARP45396_P050
RRID: AB_2047840
Goat anti-Neogenin R&D Systems Cat#: AF1079
RRID: AB_2151002
Sheep anti-SALM2 R&D Systems Cat#: AF5669
RRID: AB_1964706
Mouse anti-Tenascin-R R&D Systems Cat#: MAB1624
RRID: AB_2207001
Sheep anti-Contactin 1 R&D Systems Cat#: AF7549
Goat anti-CD200 R&D Systems Cat#: AF2724
Goat anti-ICAM5 Novus Cat#: NB100-53815
RRID: AB_829163
Goat anti- SCFR/Kit R&D Systems Cat#: AF1356
RRID: AB_354750
Mouse anti-LSAMP Developmental Studies Hybridoma Bank Cat#: 2G9
RRID: AB_2138210
Mouse anti-Trk-C R&D Systems Cat#: MAB373

	RRID: AB_2155422<br>Goat anti-Plexin-A1 R&D systems Cat#: AF4309<br>RRID: AB_10645644<br>Rat anti-HA Roche Cat#: 11867423001<br>RRID: AB 390918<br>Rabbit anti-pERM Cell Signaling Cat#: 3726<br>RRID:AB 10560513<br>Goat anti-Chicken Alexa 488 Thermo-Fisher Cat# A-11039<br>RRID: AB_2534096<br>Donkey anti-Goat Alexa 488 Thermo-Fisher Cat#: A-11055<br>RRID: AB_2534102<br>Donkey anti-Goat Alexa 647 Thermo-Fisher Cat#: A-21447<br>RRID: AB_2535864<br>Donkey anti-Rabbit Alexa 488 Thermo-Fisher Cat#: A-21206<br>RRID: AB_2535792<br>Donkey anti-Rabbit Alexa 555 Thermo-Fisher Cat#: A-31572<br>RRID: AB_162543<br>Donkey anti-Rabbit Alexa 647 Thermo-Fisher Cat#: A-31573<br>RRID: AB_2536183<br>Donkey anti-Mouse Alexa 488 Thermo-Fisher Cat#: A21202<br>RRID: AB_141607<br>Donkey anti-Mouse Alexa 555 Thermo-Fisher Cat#: A31570<br>RRID: AB_2536180<br>Donkey anti-Mouse Alexa 647 Thermo-Fisher Cat#: A-31571<br>RRID: AB_162542<br>Donkey anti-Sheep Alexa 488 Thermo-Fisher Cat# A-11015<br>RRID: AB_2534082<br>Donkey anti-Sheep Alexa 647 Thermo-Fisher Cat#: A-21448<br>RRID: AB_2535865<br>Donkey anti-Chicken Alexa 488 Jackson ImmunoResearch Cat# 703-545-155<br>RRID: AB_2340375<br>Donkey anti-Rat Alexa 647 Jackson ImmunoResearch Cat#: 712-605-153<br>RRID: AB_2340694<br>Donkey anti-Guinea Pig Cy3 Jackson ImmunoResearch Cat# 706-165-148<br>RRID: AB_2340460<br>Donkey anti-Guinea Pig Alexa 647 Jackson ImmunoResearch Cat#: 706-605-148<br>RRID: AB_2340476<br>Donkey anti-Human IgG, Fc Fragment Cy3 Jackson ImmunoResearch Cat#: 709-165-098<br>RRID: AB_2340534<br>HRP Rabbit anti-Goat IgG Thermo-Fisher Cat#: 81-1620<br>RRID: AB_2534006<br>HRP Rabbit anti-Sheep IgG Thermo-Fisher Cat#: 61-8620<br>RRID: AB_2533942<br>HRP Goat anti-Mouse IgG Thermo-Fisher Cat#: 62-6520<br>RRID: AB_2533947<br>HRP Goat anti-Rabbit IgG Thermo-Fisher Cat#: 65-6120<br>RRID: AB_2533967<br>Placental alkaline phosphatase monoclonal antibody (8B6.18) Thermo-Fisher Cat#: MA5-12694; RRID: AB_10978663<br>HRP Mouse anti-Human IgG1 Serotec Cat#: MCA514P
Validation	Most antibodies used are validated and well-accepted in the field. Information regarding available validation studies on less characterized antibodies used is carefully described in Supplementary Data 2.

Eukaryotic cell lines

Policy information about cell lines

Cell line source(s)	HEK293T from ATTC; Cat#: CRL-3216; RRID: CVCL_0063.
Authentication	Cell line was obtained from ATTC. Cell line was not authenticated.
Mycoplasma contamination	Cell lines tested negative for mycoplasma contamination.
Commonly misidentified lines<br>(See ICLAC register)	No commonly misidentified lines were used in the study.

Palaeontology

Specimen provenance	*Provide provenance information for specimens and describe permits that were obtained for the work (including the name of the issuing authority, the date of issue, and any identifying information).*
Specimen deposition	*Indicate where the specimens have been deposited to permit free access by other researchers.*
Dating methods	*If new dates are provided, describe how they were obtained (e.g. collection, storage, sample pretreatment and measurement), where they were obtained (i.e. lab name), the calibration program and the protocol for quality assurance OR state that no new dates are provided.*

☐ Tick this box to confirm that the raw and calibrated dates are available in the paper or in Supplementary Information.

Animals and other organisms

Policy information about studies involving animals; ARRIVE guidelines recommended for reporting animal research

Laboratory animals	Igsf8 cKO (B6D2;129S2-Igsf8tm1.1Osb); Riken Institute; Cat#: RBRC05637; RRID:IMSR_RBR C05637<br>Tg(Rbp4-cre)KL100Gsat; GENSATCat#: 031125-UCD; RRID:MMRRC_031125-UCD<br>C57BL/6J JAX Cat#: 000664; RRID:IMSR_JAX:000664<br>Mice were maintained in a specific pathogen-free facility under standard housing conditions with continuous access to food and water (temperature 22±2°C; 345-70% humidity). A combination of male and female mice were used in the study (1-8 weeks old), maintained on a 14h light, 10h dark light cycle from 7h to 21h.
Wild animals	Study did not involve wild animals.
Field-collected samples	Study did not involve samples collected from the field.
Ethics oversight	All animal experiments were conducted according to the KU Leuven ethical guidelines and approved by the KU Leuven Ethical Committee for Animal Experimentation (approved protocol numbers ECD P037/2016, P014/2017 and P062/2017).

Note that full information on the approval of the study protocol must also be provided in the manuscript.

Human research participants

Policy information about studies involving human research participants

Population characteristics	*Describe the covariate-relevant population characteristics of the human research participants (e.g. age, gender, genotypic information, past and current diagnosis and treatment categories). If you filled out the behavioural & social sciences study design questions and have nothing to add here, write "See above."*
Recruitment	*Describe how participants were recruited. Outline any potential self-selection bias or other biases that may be present and how these are likely to impact results.*
Ethics oversight	*Identify the organization(s) that approved the study protocol.*

Note that full information on the approval of the study protocol must also be provided in the manuscript.

Clinical data

Policy information about clinical studies

All manuscripts should comply with the ICMJEguidelines for publication of clinical research and a completedCONSORT checklist must be included with all submissions.

Clinical trial registration	*Provide the trial registration number from ClinicalTrials.gov or an equivalent agency.*
Study protocol	*Note where the full trial protocol can be accessed OR if not available, explain why.*
Data collection	*Describe the settings and locales of data collection, noting the time periods of recruitment and data collection.*
Outcomes	*Describe how you pre-defined primary and secondary outcome measures and how you assessed these measures.*

ChIP-seq

Data deposition

☐ Confirm that both raw and final processed data have been deposited in a public database such as GEO.

☐ Confirm that you have deposited or provided access to graph files (e.g. BED files) for the called peaks.

Data access links<br>*May remain private before publication.*	*For "Initial submission" or "Revised version" documents, provide reviewer access links. For your "Final submission" document, provide a link to the deposited data.*
Files in database submission	*Provide a list of all files available in the database submission.*
Genome browser session<br>(e.g. UCSC)	*Provide a link to an anonymized genome browser session for "Initial submission" and "Revised version" documents only, to enable peer review. Write "no longer applicable" for "Final submission" documents.*

Methodology

Replicates	*Describe the experimental replicates, specifying number, type and replicate agreement.*
Sequencing depth	*Describe the sequencing depth for each experiment, providing the total number of reads, uniquely mapped reads, length of reads and whether they were paired- or single-end.*
Antibodies	*Describe the antibodies used for the ChIP-seq experiments; as applicable, provide supplier name, catalog number, clone name, and lot number.*
Peak calling parameters	*Specify the command line program and parameters used for read mapping and peak calling, including the ChIP, control and index files used.*
Data quality	*Describe the methods used to ensure data quality in full detail, including how many peaks are at FDR 5% and above 5-fold enrichment.*
Software	*Describe the software used to collect and analyze the ChIP-seq data. For custom code that has been deposited into a community repository, provide accession details.*

Flow Cytometry

Plots

Confirm that:

- [x] The axis labels state the marker and fluorochrome used (e.g. CD4-FITC).
- [x] The axis scales are clearly visible. Include numbers along axes only for bottom left plot of group (a 'group' is an analysis of identical markers).
- [ ] All plots are contour plots with outliers or pseudocolor plots.
- [ ] A numerical value for number of cells or percentage (with statistics) is provided.

Methodology

Sample preparation	For initial characterization and comparison of crude MF synaptosomes and standard hippocampal synaptosomes, mouse hippocampi were dissected quickly in ice-cold Hank's Balanced Salt Solution (HBSS) and homogenized in homogenization buffer (0.32 M sucrose, 5 mM Trizma Base, 1 mM MgCl2, pH 7.4) with protease inhibitors (pepstatin A, leupeptin, aprotinin and PMSF) using a Dounce homogenizer. Homogenate was spun at 1,000 x g to pellet MF synaptosomes with nuclei and large cell debris in pellet 1 (P1), while general small synaptosomes are in the supernatant (S1). P1 and S1 fractions were loaded on Percoll gradients (Thermo-Fisher) and spun at 32,500 x g at 4°C for 7 minutes to separate MF synaptosomes and standard synaptosomes from myelin, nuclei or mitochondria, resulting in PI (crude MF) and SI (small hippocampal) synaptosome fractions. To prepare MF synaptosomes for sorting 10-12 post-natal day 28 (P28) WT mice were used per experiment. Hippocampi were dissected quickly in ice-cold HBSS and homogenized in homogenization buffer (0.32 M sucrose, 4 mM Hepes, 1 mM MgCl2, pH 7.4) with protease inhibitors (pepstatin A, leupeptin, aprotinin and PMSF) using a Dounce homogenizer. After, the homogenate was filtered through a series of cell strainers (100µm, 70 µm and 30 µm) and spun at 1,000 x g for 10 minutes at 4°C to prepare P1, which includes large MF synaptosomes, and S1. P1 was washed once by re-suspension in homogenization buffer and re-centrifuged as above. Supernatants S1 were pooled and spun at 15,000 x g for 20 minutes at 4°C to prepare post-nuclear pellet (P2) synaptosomes. P1 and P2 were re-suspended in PBS and myelin was depleted from both fractions according to the instructions of a commercial myelin-removal kit (Miltenyi Biotec), before sorting MF synaptosomes from myelin-depleted P1. After, MF synaptosomes were labeled in non-permeabilizing conditions in PBS with an anti-Nectin 3 monoclonal antibody (1:50; Hycult Biotech) directly conjugated with CF488A fluorophore (Sigma-Aldrich) after depletion of bovine serum albumin present in the antibody storage solution, using an antibody clean-up kit (Thermo-Fisher). Immediately before running the samples in the cytometer, FM4-64 (1:400; Thermo-Fisher) was added to myelin-depleted MF synaptosomes in order to label all membrane-containing material.
Instrument	BD FACS Aria III
Software	BD FACS Aria III integrated software.
Cell population abundance	From 500,000 to 1,000,000 FM4-64/Nectin 3-488-double positive particles (MF synaptosomes) were sorted on the BD FACS Aria III.
Gating strategy	FM4-64/Nectin 3-488-double positive MF synaptosomes were sorted on a BD FACS Aria III using an 85µm nozzle at 45psi. The pressure differential was set to 2 to minimize sheer stress. Nectin 3-488 was excited using a 488nm 13mW laser and detected with a 530/30 band pass filter. FM4-64 was excited using a 561nm 30mW laser and detected with a 720/40 band pass filter.

Unstained and single stained CF488A and FM4-64 synaptosomes were analyzed to calculate autofluorescence and signal above background, respectively, and Nectin 3-488 and FM4-64 gates were set accordingly. Synaptosomes were identified by back-gating on fluorescence using FSC and SSC, with SSC set on a 5 decade log scale.

☒ Tick this box to confirm that a figure exemplifying the gating strategy is provided in the Supplementary Information.

Magnetic resonance imaging

Experimental design

Design type — *Indicate task or resting state; event-related or block design.*

Design specifications — *Specify the number of blocks, trials or experimental units per session and/or subject, and specify the length of each trial or block (if trials are blocked) and interval between trials.*

Behavioral performance measures — *State number and/or type of variables recorded (e.g. correct button press, response time) and what statistics were used to establish that the subjects were performing the task as expected (e.g. mean, range, and/or standard deviation across subjects).*

Acquisition

Imaging type(s) — *Specify: functional, structural, diffusion, perfusion.*

Field strength — *Specify in Tesla*

Sequence & imaging parameters — *Specify the pulse sequence type (gradient echo, spin echo, etc.), imaging type (EPI, spiral, etc.), field of view, matrix size, slice thickness, orientation and TE/TR/flip angle.*

Area of acquisition — *State whether a whole brain scan was used OR define the area of acquisition, describing how the region was determined.*

Diffusion MRI ☐ Used ☐ Not used

Preprocessing

Preprocessing software — *Provide detail on software version and revision number and on specific parameters (model/functions, brain extraction, segmentation, smoothing kernel size, etc.).*

Normalization — *If data were normalized/standardized, describe the approach(es): specify linear or non-linear and define image types used for transformation OR indicate that data were not normalized and explain rationale for lack of normalization.*

Normalization template — *Describe the template used for normalization/transformation, specifying subject space or group standardized space (e.g. original Talairach, MNI305, ICBM152) OR indicate that the data were not normalized.*

Noise and artifact removal — *Describe your procedure(s) for artifact and structured noise removal, specifying motion parameters, tissue signals and physiological signals (heart rate, respiration).*

Volume censoring — *Define your software and/or method and criteria for volume censoring, and state the extent of such censoring.*

Statistical modeling & inference

Model type and settings — *Specify type (mass univariate, multivariate, RSA, predictive, etc.) and describe essential details of the model at the first and second levels (e.g. fixed, random or mixed effects; drift or auto-correlation).*

Effect(s) tested — *Define precise effect in terms of the task or stimulus conditions instead of psychological concepts and indicate whether ANOVA or factorial designs were used.*

Specify type of analysis: ☐ Whole brain ☐ ROI-based ☐ Both

Statistic type for inference (See Eklund et al. 2016) — *Specify voxel-wise or cluster-wise and report all relevant parameters for cluster-wise methods.*

Correction — *Describe the type of correction and how it is obtained for multiple comparisons (e.g. FWE, FDR, permutation or Monte Carlo).*

Models & analysis

n/a	Involved in the study
☐	☐ Functional and/or effective connectivity
☐	☐ Graph analysis
☐	☐ Multivariate modeling or predictive analysis

Functional and/or effective connectivity	*Report the measures of dependence used and the model details (e.g. Pearson correlation, partial correlation, mutual information).*
Graph analysis	*Report the dependent variable and connectivity measure, specifying weighted graph or binarized graph, subject- or group-level, and the global and/or node summaries used (e.g. clustering coefficient, efficiency, etc.).*
Multivariate modeling and predictive analysis	*Specify independent variables, features extraction and dimension reduction, model, training and evaluation metrics.*
